# Predictive Value of the DETECT Algorithm for Pulmonary Arterial Hypertension in Systemic Sclerosis: Findings from an Italian Observational Study

**DOI:** 10.3390/jcm14020638

**Published:** 2025-01-20

**Authors:** Stefano Stano, Claudia Iannone, Carlo D’Agostino, Maria Rosa Pellico, Livio Urso, Nicoletta Del Papa, Roberto Caporali, Fabio Cacciapaglia

**Affiliations:** 1 Rheumatology Unit, Department of Precision and Regenerative Medicine Jonian Area (DiPReMeJ), University of Bari “Aldo Moro”, 70124 Bari, Italy; stano.stefano.5@gmail.com (S.S.); livio.urso@email.it (L.U.); 2 Division of Clinical Rheumatology, ASST Gaetano Pini-CTO Institute, 20122 Milan, Italymariarosa.pellico@unimi.it (M.R.P.); roberto.caporali@unimi.it (R.C.); 3 Division of Hospital Cardiology, Cardiothoracic Department, Policlinico University Hospital, 70124 Bari, Italy; 4 Rheumatology Service, Internal Medicine Unit “F. Miulli” General Hospital, Acquaviva delle Fonti, 70021 Bari, Italy; 5 Department of Medicine and Surgery, LUM ’G. De Gennaro’, Casamassima, 70010 Bari, Italy

**Keywords:** systemic sclerosis (SSc), pulmonary arterial hypertension (PAH), DETECT algorithm, right heart catheterization (RHC)

## Abstract

**Background/Objectives:** Pulmonary arterial hypertension (PAH) is a complication of systemic sclerosis (SSc), and several screening algorithms have been proposed for the early detection of PAH in SSc. This study aimed to evaluate the predicting values of the DETECT algorithm for SSc-PAH screening in patients with SSc undergoing right heart catheterization (RHC) based on 2015 ESC/ERS echocardiographic criteria in a real-life setting. **Methods:** Patients fulfilling the 2013 ACR/EULAR classification criteria for SSc and with available data for PAH screening with the DETECT algorithm and the 2015 ESC/ERS echocardiographic criteria were retrospectively enrolled from January to June 2017 and then followed for 5 years. Baseline and annual clinical, laboratory, and instrumental data were collected. **Results**: A total of 33 out of 131 (25%) patients were selected based upon the ESC/ERS echocardiographic criteria, but 30 (23%) underwent RHC, while 51 (39%) patients with SSc were positive based on the DETECT algorithm. PAH diagnosis was confirmed in 28/30 cases (93.3%). The DETECT algorithm showed lower specificity and positive predictive value (PPV) (*p* < 0.0001) but higher sensitivity and negative predictive value (NPV) (*p* < 0.0001) than ESC/ERS criteria. Notably, patients with SSc with a negative DETECT screening at baseline had a low probability of developing PAH during a 5-year follow-up (OR 0.15, 95% CI 0.10–0.60—*p* < 0.0001). **Conclusions:** The DETECT algorithm has proven to be an easy, fast, and inexpensive tool for screening PAH in patients with SSc. Overall, a low probability of PAH using DETECT is highly predictive of a good prognosis.

## 1. Introduction

Systemic sclerosis (SSc) is a complex autoimmune disease characterized by progressive skin fibrosis and internal organ involvement, presenting a chronic and potentially progressive course but with a wide patient-to-patient variability [1]. According to recent epidemiological evidence, pulmonary involvement in the form of interstitial lung disease (ILD) and pulmonary hypertension (PH) has become the leading cause of morbidity and mortality in patients with SSc [2,3]. Data from extensive national and international registries report that pulmonary arterial hypertension (PAH) in patients with SSc ranges from 5 to 12% [4]. A recent systematic review with metanalysis concluded that the overall PAH prevalence was 6.4% (95% CI 5–8.3%), and the overall PAH incidence was 18.2 cases per 1000 person-years (95% CI 12–27.4) [5].

In 2015, the European Society of Cardiology (ESC) and the European Respiratory Society (ERS) proposed specific guidelines to standardize pulmonary arterial hypertension (PAH) diagnosis and treatment. The tricuspid regurgitation velocity (TRV) for pulmonary arterial pressure (PAP) estimation with the evaluation of other echocardiographic signs suggesting PAH has been proposed to guide the referral to the hemodynamic assessment by right heart catheterization (RHC). The gold standard measures to diagnose PAH are the mean PAP and the pulmonary capillary wedge pressure (PCWP) with resistance in pulmonary artery assessment [6]. The main drawback is that this exam is invasive and difficult to perform, except in PH referral centers [7].

Since early PAH symptoms are non-specific, even if in a high-risk population such as patients with SSc, many patients often receive a late diagnosis, showing a more compromised clinical status and severe hemodynamic features, as documented by French National Registry data [8]. Early diagnosis of PAH with screening strategies and prompt treatment with effective agents are associated with increased survival and may improve quality of life, specifically in patients with SSc [9]. Consistently, patients with SSc with PAH diagnosis made by systematic use of combined screening strategies have better clinical parameters and cardiopulmonary hemodynamics at the RHC, with a better long-term prognosis [10]. Among the proposed screening tools, the DETECT algorithm is a forward stepwise procedure designed in 2014 for patients with SSc to be referred to RHC [11]. In local rheumatology centers, patients often have limited access to experienced cardiology centers for the study of right heart diseases; therefore, a feasible tool such as DETECT can be of aid in identifying early SSc-PAH patients but also discriminating patients with a low-risk profile from those at higher risk of developing PAH.

Unfortunately, only a few studies on a limited number of patients with SSc have applied the DETECT algorithm in real-life settings [12]; moreover, to our knowledge, no studies on Italian patients with SSc are available.

Therefore, this study aimed to assess the DETECT algorithm’s predicting values in PAH diagnosis in a real-life setting of a multicenter Italian SSc cohort.

## 2. Materials and Methods

### 2.1. Study Population and Data Collection

This study evaluated patients with SSc longitudinally followed at two Italian rheumatology units with long-term experience in SSc patient management. Consecutive patients fulfilling the 2013 EULAR/ACR criteria for SSc [13] were enrolled in the present study from January to June 2017. The inclusion criteria for the analysis were age between 18 and 80, available clinical data for PAH screening according to the 2015 ESC/ERS guidelines [6], an annual follow-up visit until December 2023, and availability of RHC data for PAH diagnosis. Patients under 18 and over 80, with evidence of left ventricular dysfunction, without information on RHC, and no 5-year follow-up, were excluded.

All clinical and instrumental data of patients with SSc, including age, gender, and disease duration, were collected through medical records. The clinical assessment encompassed data regarding disease cutaneous subsets, specific serum SSc autoantibodies, comorbidities, and interstitial lung disease (ILD) at lung high-resolution computed tomography (HRCT) scans. Ongoing treatments, including glucocorticoids and immunosuppressive agents, were recorded.

This study complied with the ethical guidelines of the 1975 Helsinki Declaration and was approved by the local ethics committees (Policlinico di Bari protocol n. 5351/2017, ASST Pini CTO ID 3339, Study number 6549). All enrolled subjects gave their written informed consent to participate and to have their data used for publication, with explicit protection of their identity.

### 2.2. PH Screening Strategies

The risk of PAH development was estimated in all patients with SSc according to the 2015 ESC/ESR guidelines [6] and the DETECT algorithm [11]. The 2022 updated ESC/ERS guidelines were not applicable, as our analysis was conducted on data collected before their release [14].

The DETECT algorithm integrates a set of clinical and laboratory parameters with a two-round assessment to identify PAH early in patients with SSc [11]. Compared with traditional screening methods, which often rely only on echocardiography, the DETECT algorithm assesses the likelihood of PAH using a multifactorial approach. It preliminarily combines clinical, serological, and functional parameters and then some echocardiographic findings for a comprehensive risk assessment.

Practically, in Step 1, six non-invasive parameters are used to calculate a risk score: the ratio of % predicted forced vital capacity (FVC) to % predicted diffusion capacity for carbon monoxide (DLCO) at the pulmonary functional test, which reflects lung function and potential pulmonary involvement with information on gas exchange efficiency; serum urate for clearance from pulmonary circulation capacity; NT-proBNP as a biomarker of cardiac stress and right ventricular dysfunction; the presence of telangiectasias and anti-centromere antibody, SSc-specific parameters associated with higher PAH risk; and the presence of right axial deviation of the heart on electrocardiography as an indirect sign of right ventricular hypertrophy. If the weighted evaluation of all these variables with the appropriate algorithm yields a cumulative score ≥300 points, patients may be referred to echocardiography; otherwise, they are considered at low risk for PAH, with no indication of further evaluations. If echocardiography is recommended, in Step 2, the right atrium (RA) area and tricuspid regurgitation velocity (TRV) are incorporated to refine the risk. Through the combined evaluation of the findings of Step 1 and Step 2, which resulted in a weighted total score of ≥35 points, patients are considered at high risk of PAH, and the RHC is recommended for a definitive PAH diagnosis [11].

The 2015 ESR/ESC guidelines stratify patients with SSc as being at low, intermediate, or high risk of PAH, with potential indication to RHC, through an echocardiographic screening, which includes the evaluation of TRV, with pathological thresholds confirmed as ≤2.8 m/s low, 2.9–3.4 m/s intermediate, and >3.4 m/s high risk [6]. Further echocardiographic signs suggestive of PAH concerning the inferior vena cava and RA, interventricular septum, ventricles, and pulmonary artery morphology were also evaluated to stratify high-risk patients, as recommended [6].

### 2.3. PAH Diagnosis

Eligible patients who were positive for at least one PAH screening strategy and consented to RHC underwent this procedure to define PAH diagnosis using the 2015 ESC/ERS hemodynamic criteria [6]. Specifically, the diagnosis of PH was determined by mean pulmonary artery pressure (mPAP) > 25 mmHg and vascular resistance > 3.0 Wood units, and when the PCWP < 15 mmHg, a condition of precapillary hypertension was diagnosed. Patients whose mPAP elevation was >20 mmHg but <25 mmHg were considered borderline and tightly monitored, according to the same 2015 ESC/ERS guidelines.

Given the real-world nature of this study, it would have been unethical to subject patients with negative screening for PAH to invasive procedures such as RHC.

The entire cohort, including patients with negative screening, patients who underwent RHC, those who refused RHC, and those in whom RHC did not confirm PAH, were followed until December 2023 to evaluate their clinical outcomes and potential PAH development. Furthermore, patients with SSc with negative baseline screening were reassessed annually for PAH with the 2015 ESC/ERS guidelines and the DETECT algorithm during the follow-up period and underwent RHC if appropriate.

### 2.4. Statistical Analysis

Variables are reported as means with standard deviations (SD), medians with interquartile ranges (IQR), or absolute numbers with percentages, as appropriate. The D’Agostino–Pearson test was used to check for data distribution. Continuous variables were compared using paired *t*-test or Mann–Whitney test when appropriate. Categorical variables were compared using Fisher’s exact test. According to 2015 ESC/ERS guidelines and the DETECT algorithm, the risk of having PAH was estimated as an odds ratio (OR) and 95% confidence interval (CI), with relative *p*-values. The Kaplan–Meier analysis and the log-rank test with hazard ratio (HR) and a 95% CI were used to compare the 5-year rate of PAH development. Statistical analysis of the data was carried out using GraphPad Prism software (v. 9.5.1); a *p*-value < 0.05 was considered statistically significant.

## 3. Results

At the enrollment 131 patients with SSc (116 female (88.5%), with a median (IQR) age of 64 (54–72) years old and a median (IQR) disease duration of 9 (6–14) years, were included in this study, and relevant clinical–demographic features are reported in Table 1. Of note, 110 (84%) patients had limited cutaneous involvement, and 21 (16%) presented a diffuse cutaneous involvement. All patients with SSc were anti-nuclear antibodies (ANA) positive, 47 (35.9%) had anti-centromeric proteins (CENP) antibodies, and 57 (43.5%) had anti-Topoisomerase I. The presence of ILD was observed in 78 (59.5%) patients. Comorbidities were present in 58 (44.3%) cases. Treatment with oral corticosteroids was administered in 60.3% of patients with SSc, and immunosuppressive therapy was administered in 63.3% of cases.

**Figure 1:**
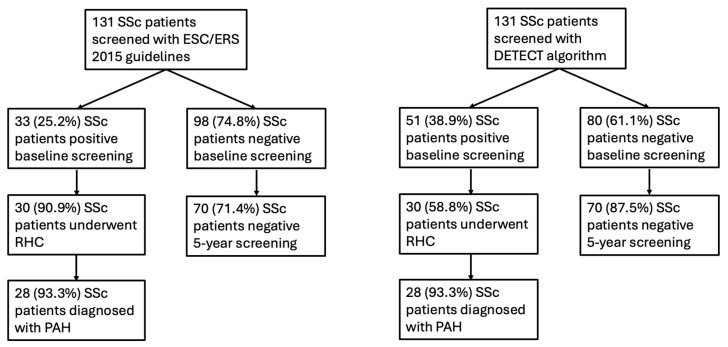
Flow chart of PAH screening in patients with systemic sclerosis (SSc) with the ESC/ERS 2015 guidelines and the DETECT algorithm. Abbreviations: SSc, systemic sclerosis; RHC, right heart catheterism; PAH, pulmonary arterial hypertension; ESC/ERS, European Society of Cardiology/European Respiratory Society.

Figure 1 shows the flowchart of ESC/ERS 2015 and DETECT strategies used for the PAH screening in this study. Fifty-two patients with SSc (40%) were positive for at least one screening strategy. Specifically, after echocardiographic assessment, according to the 2015 ESC/ERS guidelines, 33 out of 131 (25%) patients were at high risk for PAH. While applying Step 1 of DETECT screening, 91 out of 131 (69%) patients required an echocardiographic evaluation, and 51 out of 91 patients (56%) were positive at Step 2. A total of 30 patients with SSc consented to undergo RHC, and PAH diagnosis was confirmed in 28 cases (93%). Ultimately, 85% (28/33) of patients selected via ESC/ERS 2015 screening were confirmed to have PAH at RHC, while at DETECT screening, 54.9% (28/51) satisfied the diagnosis of PAH by RHC. The clinical characteristics of patients with SSc diagnosed with PAH are summarized in Table 2. No further patients underwent RHC and received PAH diagnosis during the 5-year follow-up.

**Figure 2:**
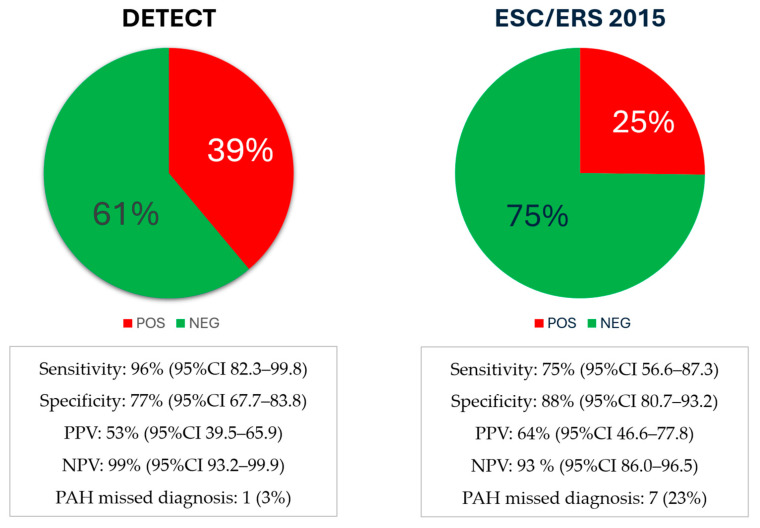
Predictive screening values with the DETECT algorithm and the 2015 ESC/ERS guidelines in patients with SSc for PAH diagnosis. Abbreviations: PPV, positive predictive value; NPV, negative predictive value; SSc, systemic sclerosis; RHC, right heart catheterism; PAH, pulmonary arterial hypertension; ESC/ERS, European Society of Cardiology/European Respiratory Society.

Both algorithms were effective PAH screening strategies, able to discriminate patients with SSc at high risk for PAH development (DETECT algorithm: OR 88.9, 95% CI 14.3–924.2, *p* < 0.0001; 2015 ESC/ERS guidelines: OR 22.7, 95% CI 8.2–59.7, *p* < 0.0001). The DETECT algorithm had higher sensitivity and negative predictive value (NPV) (*p* < 0.0001) but lower specificity and positive predictive value (PPV) (*p* < 0.0001) for PAH diagnosis compared to the 2015 ESC/ERS guidelines [Figure 2]. Differently, in only one case (3%), negative at DETECT screening but at high risk for 2015 ESC/ERS guidelines, a PAH diagnosis was confirmed at RHC. Interestingly, patients with SSc at high risk for PAH with at least one screening strategy, not consenting to undergo RHC, had the lowest survival rate.

During the follow-up, a total of 24 patients with SSc died, and 6 of them died from complications of PAH, with a 5-year survival rate of 81.7%. Among the 18 patients with SSc without PAH who died, 5 presented with complications from SARS-CoV2 infection, 6 had evolution of ILD involvement, 6 had cancer, and 1 died from a car accident. Of those 79 patients with SSc negative at baseline for PAH screening with both strategies, 70 (88.6%) were still alive, and none of them developed PAH [Figure 1].

Differentiating patients with SSc with or without PAH, a 5-year survival of 22 out of 28 (78.6%) in SSc-PAH patients, compared to 85 out of 103 (82.5%) in patients without PAH, was observed, with a difference that did not achieve statistical significance (*p* = 0.78).

**Figure 3:**
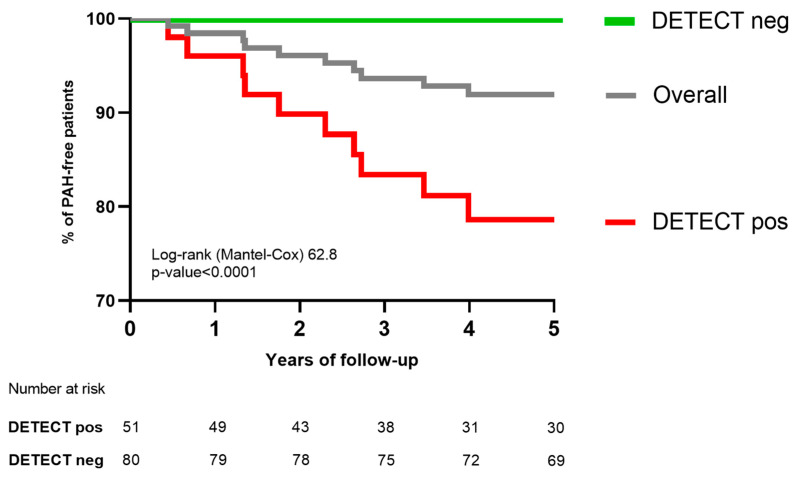
Kaplan–Meier analysis for PAH development in patients with SSc screened with the DETECT algorithm.

Notably, patients with SSc with a negative DETECT screening did not develop subsequent PAH, as demonstrated via Kaplan–Meier analysis [Figure 3].

## 4. Discussion

We studied the effectiveness of the DETECT algorithm and the 2015 ESC/ERS echocardiographic criteria for assessing PAH risk in patients with SSc in a real-life setting. A total of 30 out of 33 patients with SSc (91%) with positive ESC/ERS echocardiographic screening consented and were eligible according to RHC cardiologic criteria. Among them, PAH diagnosis was confirmed in 28 cases (93%). The DETECT algorithm showed significantly higher sensitivity and NPV. Patients with SSc with negative DETECT screening had a very low probability of developing PAH after 5 years of follow-up.

Despite significant variability in time of occurrence from SSc diagnosis, PAH is a relatively frequent complication of SSc, with an estimated prevalence between 10% and 20%, depending on follow-up duration [15]. Early diagnosis and effective management of SSc-PAH still represent an unmet clinical need nowadays, as this condition is the cause of death in around 30% of patients with SSc [16,17], with, on average, a diagnostic delay between the onset of symptoms and diagnosis of 2–4 years [18]. In our study, we observed that the prevalence of SSc-PAH in the Italian SSc cohort was 21.4% after a median disease duration of 9 years and demonstrated the effectiveness of regular screening with the DETECT algorithm to identify patients with SSc at high risk of PAH development in a real-world setting. We found that the DETECT algorithm had a high sensitivity and negative predictive value in screening patients with SSc, strongly supporting its use in clinical practice.

According to a recent survey [19], British rheumatologists recognize the importance of screening for PAH in patients with SSc despite certain limitations and variability in the methodology. The most frequent challenges were the difficulty of interpreting results from other hospitals and long waiting lists for diagnostic tests. Access to critical investigations, clinician education, multidisciplinary meetings, and a better understanding of available screening algorithms were proposed as potentially effective solutions [20]. The hub-and-spoke model, successfully implemented in some American rural hospitals, may overcome these limits by centralizing essential resources [20]. This organizational design provides access to patients from peripheral hospitals to tertiary consultations with physicians of healthcare institutions through a national integrated service [21]. Our data confirm the availability of an accessible tool that may guarantee adequate performance in SSc-PAH screening programs. Screening of PAH in patients with SSc should be performed according to standardized protocols able to predict the development of this comorbidity early. The updated (2022) ESC/ERS guidelines for PH recommend an annual evaluation of PAH risk in all patients with SSc, specifically in those with >3 years of disease duration, FVC > 40%, and DLCO <60% [13]. The DETECT algorithm was adequate, with good performance in the asymptomatic stage, also among individuals with a DLCO of ≥60%, even when PAH was defined according to the last updated ESC/ERS hemodynamic criteria [22,23]. A recent study comparing different screening strategies for PAH showed that the frequency of PAH diagnosis has increased by 1.8-fold with updated ESC/ERS definitions. However, algorithms for PAH screening appear less sensitive for the new PAH diagnostic cut-off. Therefore, the multimodal/algorithmic approach is strongly advised, representing the best option for predicting PAH risk [24]. Of note, in our SSc cohort, for those patients with a negative DETECT algorithm (Step 1), the risk of PAH appeared to be negligible as no cases of PAH were observed after 5 years of follow-up, with 100% NPV. Therefore, despite having carried out annual echocardiography in all patients with SSc according to 2015 ESC/ERS guidelines, those patients not advised to undergo echocardiography according to the DETECT algorithm could have avoided this exam.

Indeed, in Step 1 of the DETECT algorithm, among all the six parameters that contribute to the calculation of the composite risk score for PAH in patients with SSc, recent studies analyzing the algorithm showed that NT-proBNP and % predicted DLCO were the most weighted and influential parameters in predicting PAH [22,25,26]. The NT-proBNP levels strongly predict PAH as a direct biomarker of right ventricular strain, a hallmark of PAH, and reflect cardiac stress and overload, often caused by elevated pulmonary vascular resistance. On the other hand, a low % predicted DLCO, an established indicator of impaired gas exchange, typically reflects pulmonary vascular abnormalities. If severely reduced, % predicted DLCO is a distinguishing feature of PAH from other complications of SSc, such as ILD, often being considered the strongest pulmonary functional predictor of PAH risk.

Our results confirm the importance of regular screening for PAH in patients with SSc according to specific protocols, such as the DETECT algorithm. This screening tool encompasses different parameters, evaluating lung function and gas exchange, cardiac strain and hemodynamics, and SSC-specific clinical and laboratory features. Its weighted multifaceted approach can be applied for screening in all patients with SSc, ensuring high sensitivity to detect PAH early. Twenty-eight (21.4%) PAH diagnoses were confirmed at RHC in our SSc cohort during five years of follow-up. Only one patient with negative DETECT screening had PAH (NPV 98.7%) compared to six cases with the 2015 ESC/ERS guidelines (NPV 92.9%). Notably, early systematic screening of PAH in patients with SSc seems to positively affect the healthcare system too, as economic analysis of screening modalities for early PAH development in patients with SSc carried out in French, Australian, and Belgian cohorts demonstrated that screening programs are also cost saving [27,28]. According to a study performed at the SSc unit for PAH of the Ghent University Hospital, the average cost per patient attending the screening program was lower when annual echocardiography according to the 2015 ESC/ERS guidelines in combination with the DETECT algorithm was carried out [29]. Our data support the findings that using the DETECT algorithm for screening SSc-PAH may save a relevant number of echocardiographic examinations, consequently reducing costs. Furthermore, we confirmed the feasibility of this algorithm in routine clinical practice, as it can be completed quickly in a few minutes during outpatient visits. Therefore, we suggest the systematic use of the DETECT algorithm in all patients with SSc, regardless of disease duration, NT-proBNP, and DLCO levels, to minimize the number of PAH missed diagnoses but also to stratify PAH risk and management in patients with SSc according to the results of DETECT algorithm (i.e., indication to echocardiography for Step 2 examination only if indicated).

Some shortcomings of our study must be recognized. Most ESC/ERS echocardiographic screening-positive patients received an indication for RHC, and according to clinical practice, some patients denied their consent to RHC. Even if all patients were tightly followed up, no specific PAH treatment could be applied in patients not performing an RHC, and some patients with an indication of RHC may have died without a potential diagnosis of PAH. On the other hand, the longitudinal analysis for 5 years confirmed the usefulness of DETECT in discriminating against patients with SSc who do not need to undergo RHC and can, therefore, be adequately monitored by rheumatologists.

## 5. Conclusions

The present study confirms that the DETECT algorithm is a valid screening method with which to exclude PAH in patients with SSc, superior to the strategy of the 2015 ESC/ERS guidelines. Due to its higher sensitivity and NPV, it can reduce the number of unnecessary echocardiographic examinations for patients with a negative outcome after Phase 1 and reduce missed diagnoses after Phase 2. Notably, in all patients with SSc with negative DETECT screening at baseline, the subsequent development of PAH is improbable, as observed after 5 years of follow-up. Further studies on a more extensive series could confirm the performance of the DETECT algorithm in the daily clinical practice of Scleroderma Units with broader follow-up.

## Figures and Tables

**Table 1:** Demographic, clinical, and serologic characteristics of enrolled patients with SSc.

Characteristics	SSc Patients (*n* = 131)
Female, *n*. (%)	116 (88.5)
Age, years (median, IQR 25–75)	64 (54–72)
Disease duration, years (median, IQR 25–75)	9 (6–14)
Follow-up duration years (median, IQR 25–75)	9 (8–12)
Cutaneous subsets—limited/diffuse, *n* (%)	110/21 (84/16)
ANA positive, *n*. (%)	131 (100)
Anti-CENP antibodies, *n*. (%)	47 (35.9)
Anti-TopoI antibodies, *n*. (%)	57 (43.5)
Other ENA *, *n*. (%)	9 (8)
ENA negative, *n*. (%)	18 (13.7)
ILD presence at lung HR CT scan, *n*. (%)	78 (59.5)
Digital ulcers, *n*. (%)	56 (42.7)
Esophageal manifestations, *n*. (%)	52 (39.7)
Diabetes mellitus, *n*. (%)	9 (6.9)
Arterial hypertension, *n*. (%)	37 (28.2)
Chronic kidney disease, *n*. (%)	7 (5.3)
Gastrointestinal manifestations, *n*. (%)	33 (25.2)
Chronic obstructive pulmonary disease, *n*. (%)	9 (6.9)
Smoking—actual/former, *n*. (%)	13/16 (9.9/12.2)
Treatment with corticosteroids, *n*. (%)	79 (60.3)
Immunosuppressive therapy, *n*. (%)	83 (63.3)
Indication to RHC by 2015 ESC/ERS screening, *n*. (%)	33 (25.2)
Indication to RHC by DETECT screening, *n*. (%)	51 (38.9)
PAH diagnosis at RHC, *n*. (%)	28 (21.4)

Abbreviations: IQR, interquartile range; ANA, antinuclear antibodies; CENP, centromeric proteins; TopoI, Topoisomerase I; ILD, interstitial lung disease; HR CT, high-resolution computed tomography; RHC, right heart catheterization; PAH, pulmonary arterial hypertension; ESC/ERS, European Society of Cardiology/European Respiratory Society. * SSa/Ro, Th/To, RNA-polymerase III, Pm-Scl.

**Table 2:** Demographic, clinical, and serologic characteristics of SSc-PAH patients.

Characteristics	SSc-PAH Patients (*n* = 28)
Female, *n*. (%)	26 (92.9)
Age, years (median, IQR 25–75)	69 (63–76)
Disease duration, years (median, IQR 25–75)	11 (7–18)
Follow-up duration years (median, IQR 25–75)	10 (9–12)
Subset Limited, *n* (%)	21 (75)
ANA positive, *n*. (%)	28 (100)
Anti-CENP antibodies, *n*. (%)	9 (32.1)
Anti-TopoI antibodies, *n*. (%)	11 (39.3)
Other ENA *, *n*. (%)	1 (3.6)
ENA negative, *n*. (%)	7 (25)
ILD presence at lung HR CT scan, *n*. (%)	21 (75)
Digital ulcers, *n*. (%)	15 (53.6)
Esophageal manifestations, *n*. (%)	9 (32.1)
Diabetes mellitus, *n*. (%)	2 (7.1)
Arterial hypertension, *n*. (%)	8 (28.6)
Chronic kidney disease, *n*. (%)	2 (7.1)
Gastrointestinal manifestations, *n*. (%)	4 (14.3)
Chronic obstructive pulmonary disease, *n*. (%)	1 (3.6)
Smoking—actual/former, *n*. (%)	0/5 (0/17.8)
Treatment with corticosteroids, *n*. (%)	19 (67.9)
Immunosuppressive therapy, *n*. (%)	18 (64.3)
RHC positive by 2015 ESC/ERS screening, *n*. (%)	21 (75)
RHC positive by DETECT screening, *n*. (%)	27 (96.4)

Abbreviations: IQR, interquartile range; CENP, centromeric proteins; TopoI, Topoisomerase I; ILD, interstitial lung disease; HR CT, high-resolution computed tomography; RHC, right heart catheterization; PAH, pulmonary arterial hypertension; ESC/ERS, European Society of Cardiology/European Respiratory Society. * SSa/Ro, Th/To, RNA-polymerase III, Pm-Scl.

## Data Availability

All relevant data for this study are included in the article or available upon reasonable request to the corresponding author.
